# Probiotic Bacillus Spores Protect Against Acetaminophen Induced Acute Liver Injury in Rats

**DOI:** 10.3390/nu12030632

**Published:** 2020-02-27

**Authors:** Maria Adriana Neag, Adrian Catinean, Dana Maria Muntean, Maria Raluca Pop, Corina Ioana Bocsan, Emil Claudiu Botan, Anca Dana Buzoianu

**Affiliations:** 1 Department of Pharmacology, Toxicology and Clinical Pharmacology, Iuliu Hatieganu University of Medicine and Pharmacy, Cluj-Napoca 400337, Romania; maria.neag@umfcluj.ro (M.A.N.); raluca_parlog@yahoo.com (M.R.P.); corinabocsan@yahoo.com (C.I.B.); abuzoianu@umfcluj.ro (A.D.B.); 2 Department of Internal Medicine, Iuliu Hatieganu University of Medicine and Pharmacy, Cluj-Napoca 400006, Romania; 3 Department of Pharmaceutical Technology and Biopharmaceutics, Iuliu Hatieganu University of Medicine and Pharmacy, Cluj-Napoca 400010, Romania; dana@tbs.ubbcluj.ro; 4 County Emergency Hospital Cluj-Napoca, Cluj-Napoca 400006, Romania; botanemil@gmail.com

**Keywords:** *Bacillus* spores, acute hepatotoxicity, acetaminophen, tight junction, inflammation

## Abstract

Acetaminophen (APAP) is one of the most used analgesics and antipyretic agents in the world. Intoxication with APAP is the main cause of acute liver toxicity in both the US and Europe. Spore-forming probiotic bacteria have the ability to resist harsh gastric and intestinal conditions. The aim of this study was to investigate the possible protective effect of *Bacillus* (B) species (sp) spores (*B. licheniformis, B. indicus, B. subtilis, B. clausii, B. coagulans*) against hepatotoxicity induced by APAP in rats. A total of 35 rats were randomly divided into seven groups: group I served as control; group II received silymarin; group III received MegaSporeBiotic^TM^ (MSB); group IV received APAP and served as the model of hepatotoxicity; group V received APAP and silymarin; group VI received APAP and MSB; group VII received APAP, silymarin and MSB. The livers for histopathological examination and blood samples were collected on the last day of the experiment. We determined aspartate aminotransferase (AST), alanine aminotransferase (ALT) and total antioxidant capacity (TAC) levels and zonula occludens (ZO-1), tumor necrosis factor α (TNF-α) and interleukin 1*β* (IL-1*β*) expression. APAP overdose increased AST and ALT. It slowly decreased TAC compared to the control group, but pretreatment with silymarin and MSB increased TAC levels. Elevated plasma concentrations were identified for ZO-1 in groups treated with APAP overdose compared with those without APAP or receiving APAP in combination with silymarin, MSB or both. The changes were positively correlated with the levels of other proinflammatory cytokines (TNF-α, IL-1*β*). In addition, histopathological hepatic injury was improved by preadministration of MSB or silymarin versus the disease model group. *Bacillus* sp spores had a protective effect on acute hepatic injury induced by APAP. Pretreatment with MSB resulted in a significant reduction in serum AST, ALT, TNF-α, IL-1*β*, ZO-1, TAC and also hepatocyte necrosis, similar to the well-known hepatoprotective agent—silymarin.

## 1. Introduction

The liver is the main organ involved in maintaining the body’s homeostasis. Many chemical compounds (including medicines) and herbal remedies, when used in overdose quantities, may cause liver damage [1]. Acetaminophen (N-acetyl-p-aminophenol or paracetamol; APAP), an over-the-counter drug, is one of the most used analgesics and antipyretic agents in the world [2]. It is known that in high doses, APAP may lead to acute liver failure [3]; intentional or unintentional intoxication with APAP is the main cause of acute liver toxicity in both the United States and Europe [2]. APAP, at the therapeutic dose, is metabolized mainly by glucuronidation and sulfation (phase II reactions) to nontoxic metabolites in the liver. A minor fraction of the therapeutic dose is oxidized by the CYP450 hepatic enzymes to the reactive metabolite N-acetyl-p-benzoquinone-imine (NAPQI). When APAP is taken in high doses, the amount of NAPQI increases significantly, which depletes hepatic glutathione (GSH) storage and results in increased oxidative stress and mitochondrial dysfunction with decreased adenosine triphosphate (ATP; e.g., mitochondrial dysfunction, oxidative stress, inflammatory reactions) [4,5,6,7]. Moreover, cell damage is also the consequence of activating mitogen-activated protein kinase (MAPK), c-Jun-N-terminal kinase (JNK) or nuclear DNA fragmentation [8].

Probiotics have been shown to have beneficial effects in several ailments, from gastrointestinal disorders (inflammatory bowel diseases, liver diseases) to allergy, metabolic disorders and cancer. These effects are a consequence of restoring the balance of gut microbiota (commensal vs. pathogenic bacteria), maintaining the integrity of the intestinal barrier, reducing the production of toxic products and improving the liver function [9,10,11]. The beneficial effect of probiotics may be due to the inhibition of growth of harmful bacteria by the production of free fatty acids, hydrogen peroxide and antimicrobial peptides [12,13].

*Bacillus* species (sp) are characterized by a high level of resistance to physical and chemical agents that are normally considered harmful to microorganisms (heat, toxic chemicals, radiation) [14]. Moreover, spores have a greater resistance to technological stress and storage compared to vegetative/active probiotics. They also have the ability to resist harsh gastric and intestinal conditions (bile acids, digestive enzymes, pH) [15]. Thus, spore-forming probiotic bacteria are considered a very good alternative solution to replace *Bifidobacterium* and *Lactobacillus* strains, which have the disadvantage of low stability [16,17]. The aforementioned advantages of using *Bacillus* can explain recent efforts to open up new perspectives on the use of spore-based probiotics, which exhibit similar stability to other pharmaceutical drugs used for conventional treatment of many diseases [18].

This study was performed to evaluate the possible protective effect of *Bacillus* sp. spores (*B. licheniformis, B. indicus, B. subtilis, B. clausii, B. coagulans*) on acute hepatic injury induced by APAP overdose in rats.

## 2. Materials and Methods 

### 2.1. Drugs and Chemicals

We used MegaSporeBiotic^TM^ (MSB) probiotic capsules (Microbiome Labs, Saint Augustine, FL, USA) and two standard commercial compounds, silymarin (150 mg/tablet) and APAP (500 mg/tablet; uncoated tablets). All products were purchased from a public pharmacy and administered as a suspension with 1% carboxymethylcellulose (CMC) as the vehicle. MSB is a probiotic blend of spores from five *Bacillus* species (*B. licheniformis, B. indicus, B. subtilis, B. clausii and B. coagulans*). 

### 2.2. Animals 

Charles River Wistar white male rats (*n* = 35) weighing between 250 and 280 g were obtained from the Center for Experimental Medicine and Practical Skills of the university. The working animal protocol was revised and approved by the Ethics Committee of Iuliu Haţieganu University of Medicine and Pharmacy, nr. 12101/02.05.2018. 

The rats were kept in cages in a clean room with 12 h light/dark cycles and a temperature of 22 ± 2 °C. The animals were acclimated under these conditions for two days prior to starting the experiment. Specific regulations and amendments from this study were from the "Guiding Principles in the Use of Animals in Toxicology" adopted by the Society of Toxicology (Reston, VA, USA) and the national law regarding the protection of animals used for scientific research.

### 2.3. Experimental Design

A total of 35 rats were randomly divided into seven groups (*n* = 5/group): group I served as control and received only the vehicle, 1% CMC; group II received silymarin (100 mg/kg/day); group III received MSB (1 × 10^9^ CFU/day); group IV received APAP (2 g/kg) and served as the model of hepatotoxicity; group V received APAP (2 g/kg) and silymarin (100 mg/kg/day); group VI received APAP (2 g/kg) and MSB (1 × 10^9^ CFU/day); group VII received APAP (2 g/kg), silymarin (100 mg/kg/day) and MSB (1 × 10^9^ CFU/day). All of the substances were suspended in 1% CMC. Animals were fed rat chow ad libitum and had free access to tap water.

CMC, silymarin and MSB were administered orally through a feeding tube daily for 12 days. Groups IV, V, VI and VII received a single dose of APAP suspended in 1% CMC on day 11, also administered orally through a feeding tube. 

Blood was collected from the retro-orbital sinus plexus under mild ether anesthesia (periorbital method) on the last day of the experiment (48 h after receiving APAP; Day 13). After coagulation, the serum was separated by centrifuging at 4000 rpm for 15 min; the serum was stored at −20 °C for further biochemical analysis.

Serum aspartate aminotransferase (AST) and alanine aminotransferase (ALT) were measured by an automatic biochemical analyzer according to the manufacturer’s protocols; total antioxidant capacity (TAC) using a validated high-throughput liquid chromatography (HPLC) tandem mass spectrometry analytical method previously described by Erel [19], TNF-α, IL-1*β* and zonula occludens (ZO-1) were measured using ELISA technique (Stat Fax 303 Plus Microstrip Reader, Minneapolis, USA). Their detection and quantification were performed using commercially available kits (TNF-α and IL-1β ABTS ELISA development kits; PeproTech EC, Ltd., London, UK; and TJP1 ELISA kit; Elabscience, Houston, TX, USA). Animals were sacrificed with xylazine/ketamine overdose.

### 2.4. Histopathological Examination 

At the end of the study, the liver of each animal was removed and excised tissue sections were preserved in 10% formaldehyde, dehydrated in graduated ethanol and embedded in paraffin. Embedded liver tissues were cut into 7 μm sections using a microtome (RM 2145; Leica, Wetzlar, Germany) then mounted on glass slides. Slides were stained with hematoxylin-eosin for histological evaluation. The histological sections were examined using a Leica DM2500 LED microscope and the images were captured using a Leica DMC2900 (Leica Microsystems Ltd., Heerbrugg, Switzerland) camera which was connected to the microscope.

### 2.5. Statistical Analysis

All data were presented as mean ± standard deviation (SD). The significance between the different groups was analyzed by one-way ANOVA. SPSS 10.0 statistical software (SPSS Inc., Chicago, IL, USA) was used for all statistical analyses. A *p*-value < 0.05 was considered statistically significant.

## 3. Results

### 3.1. Effect of MegaSporeBiotic™ and Silymarin on Liver Functions 

**Figure 1:**
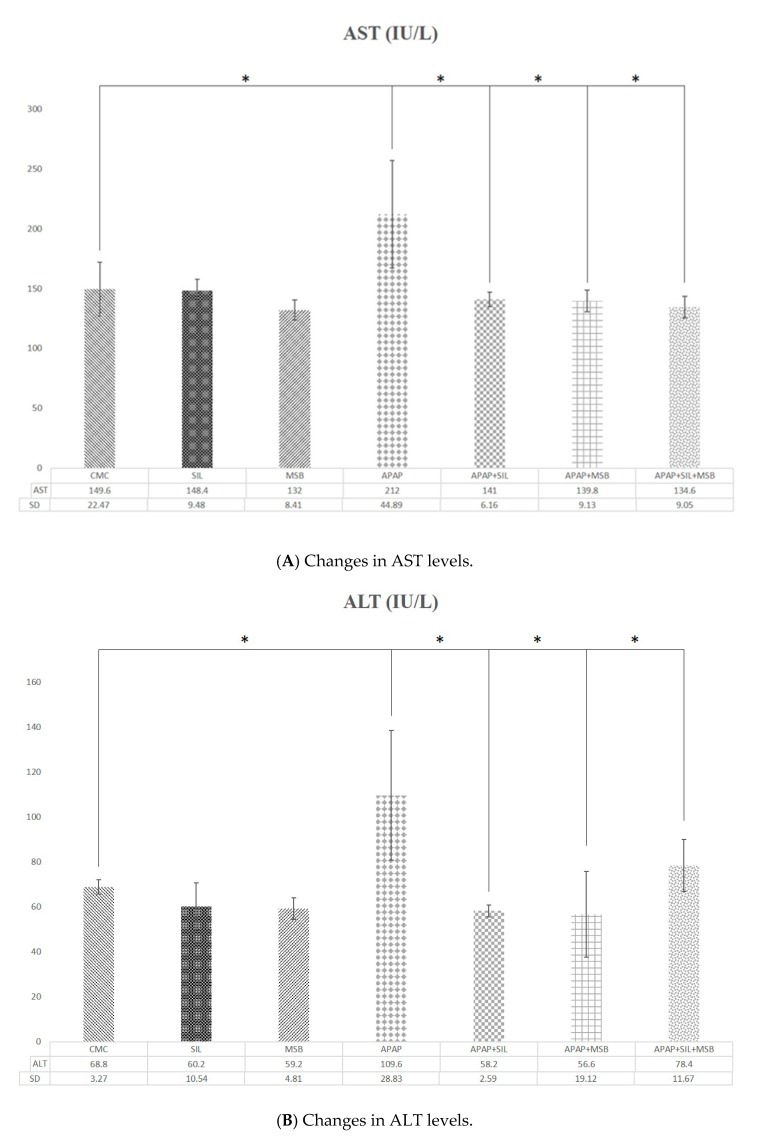
Changes in AST (dotted) and ALT (lined) levels. Rats were treated with 1% CMC, APAP, MSB or SIL either alone or in various combinations (as indicated). Periorbital blood was collected on Day 13, and the serum was tested for AST and ALT levels. * Significant difference (*p* < 0.05) between groups; data were analyzed using one-way ANOVA. Abbreviations: ALT, alanine aminotransferase; AST, aspartate aminotransferase; APAP, N-acetyl-p-aminophenol or paracetamol (acetaminophen); CMC, carboxymethylcellulose; MSB, MegaSporeBiotic™; SIL, silymarin.

Administration of APAP caused a significant elevation in serum liver enzymes (ALT, AST) (ALT, *p* = 0.017, AST, *p* = 0.007; group I vs. group IV). Pretreatment with silymarin (ALT, *p* = 0.009; AST, *p* = 0.016; group V vs. group IV), MSB (ALT, *p* = 0.007; AST, *p* = 0.013; group VI vs. group IV) or both (ALT, *p* = 0.04; AST, *p* = 0.015; group VII vs. group IV) significantly alleviated the hepatotoxic effect of APAP (Figure 1A,B). No significant differences were found between rats treated with silymarin and rats treated with MSB or both MSB and silymarin.

### 3.2. Effect of MegaSporeBiotic™ and Silymarin on Inflammation and Oxidative Stress

**Figure 2:**
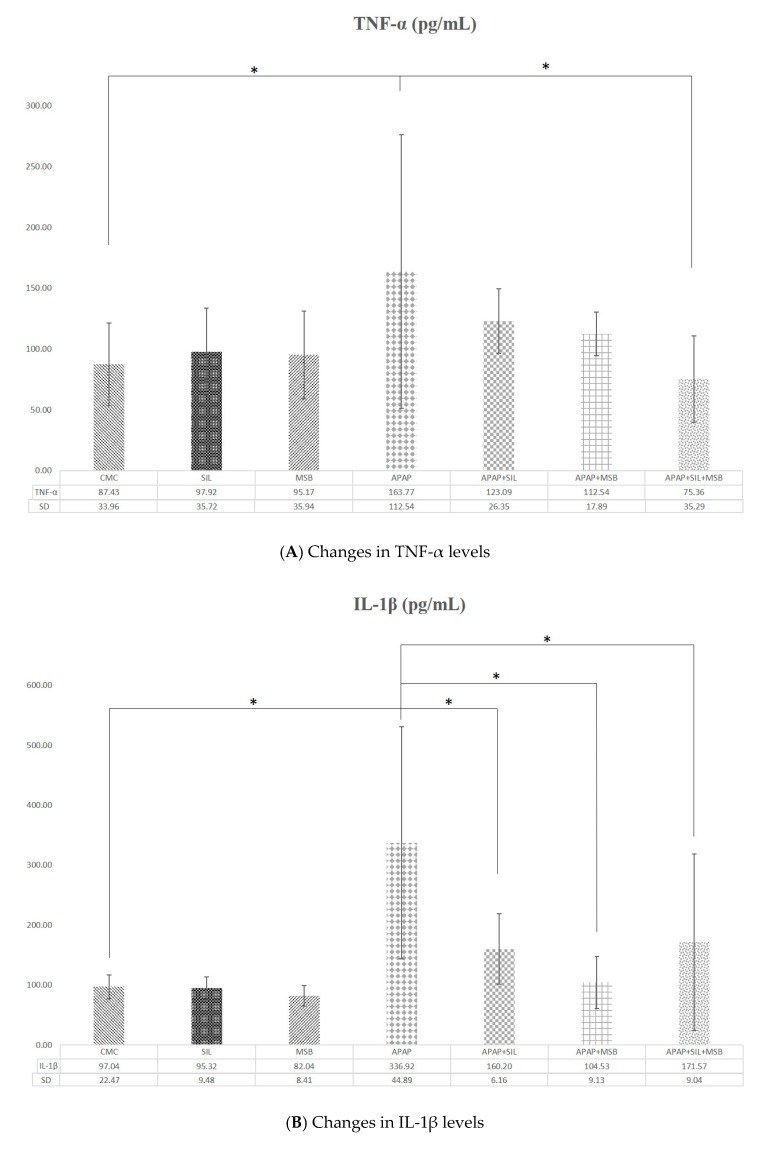
Rats were treated with 1% CMC, SIL, MSB, APAP, APAP + SIL, APAP + MSB or APAP + SIL + MSB. Periorbital blood was collected on Day 13, and the serum was tested for TNF-α (**A**) and IL-1β (**B**) levels. * Significant difference (*p* < 0.05) between groups; data were analyzed using one-way ANOVA. Abbreviations: APAP, N-acetyl-p-aminophenol or paracetamol (acetaminophen); CMC, carboxymethylcellulose; IL-1β, interleukin 1β; MSB, MegaSporeBiotic™; SIL, silymarin; TNF-α, tumoral necrosis factor α.

APAP determined marked an increase in the levels of proinflammatory cytokines TNF-α and IL-1*β* compared with the control group (*p* < 0.05). MSB and silymarin significantly decreased inflammation (Figure 2A,B).

**Figure 3:**
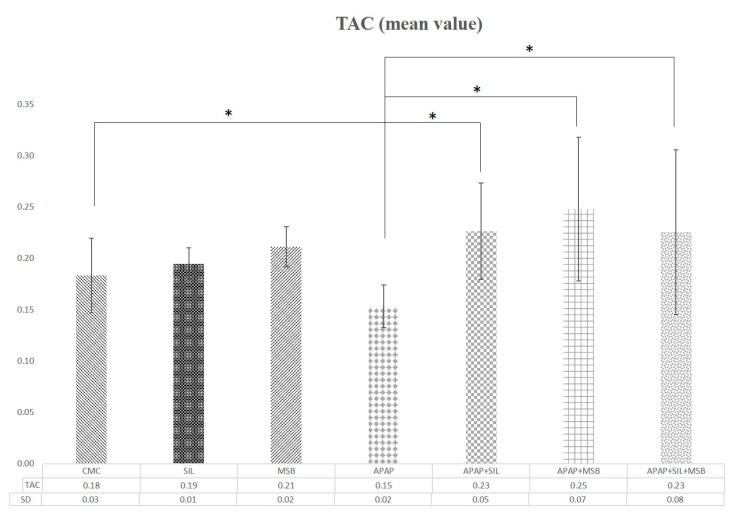
Changes in TAC levels. Rats were treated with 1% CMC, APAP, MSB or SIL either alone or in various combinations (as indicated). Periorbital blood was collected on Day 13, and the serum was tested for TAC levels. * Significant difference (*p* < 0.05) between groups; data were analyzed using one-way ANOVA. Abbreviations: APAP, N-acetyl-p-aminophenol or paracetamol (acetaminophen); CMC, carboxymethylcellulose; MSB, MegaSporeBiotic™; TAC, total antioxidant capacity; TRL, Trolox; SIL, silymarin.

To investigate the antioxidant effect of MSB, the levels of TAC were determined in rats. APAP treatment significantly decreased the TAC compared with the control group (*p* = 0.012). MSB and silymarin administered resulted in a significant increase in TAC (Figure 3).

### 3.3. Histopathology

**Figure 4:**
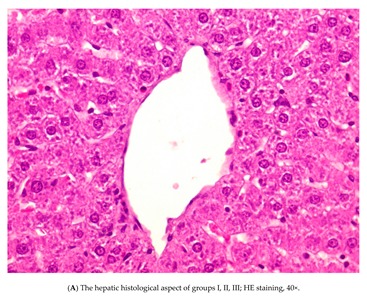

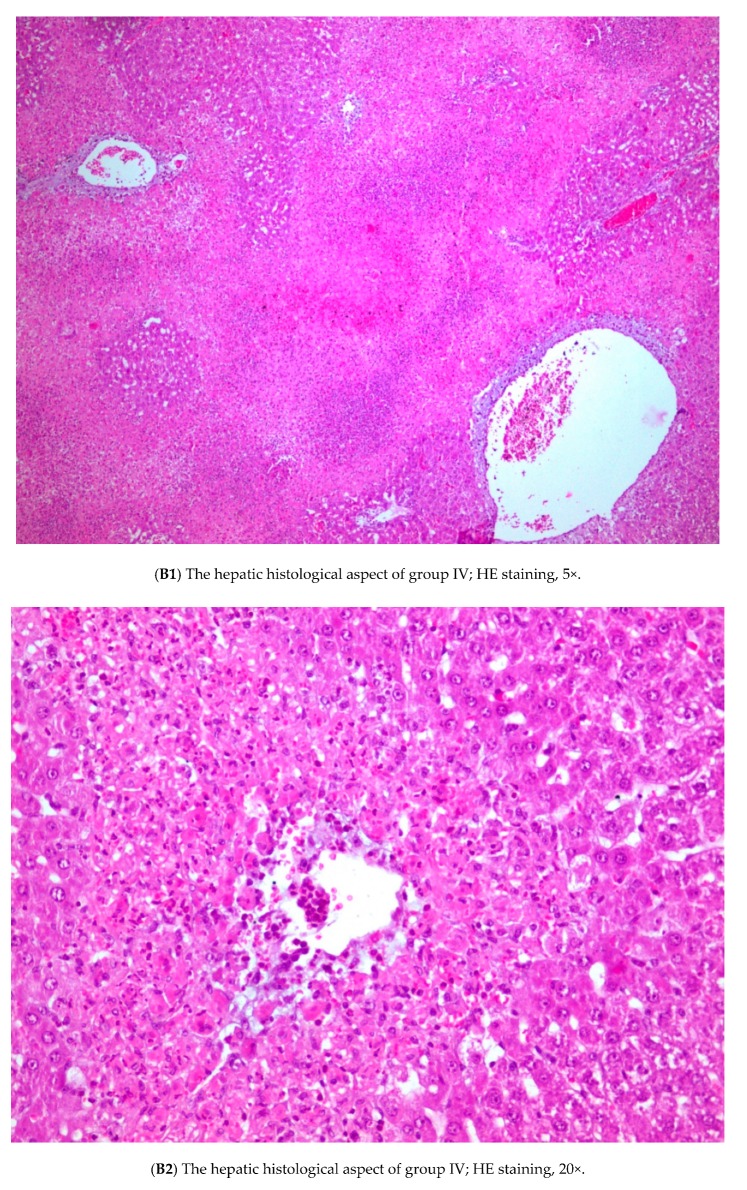

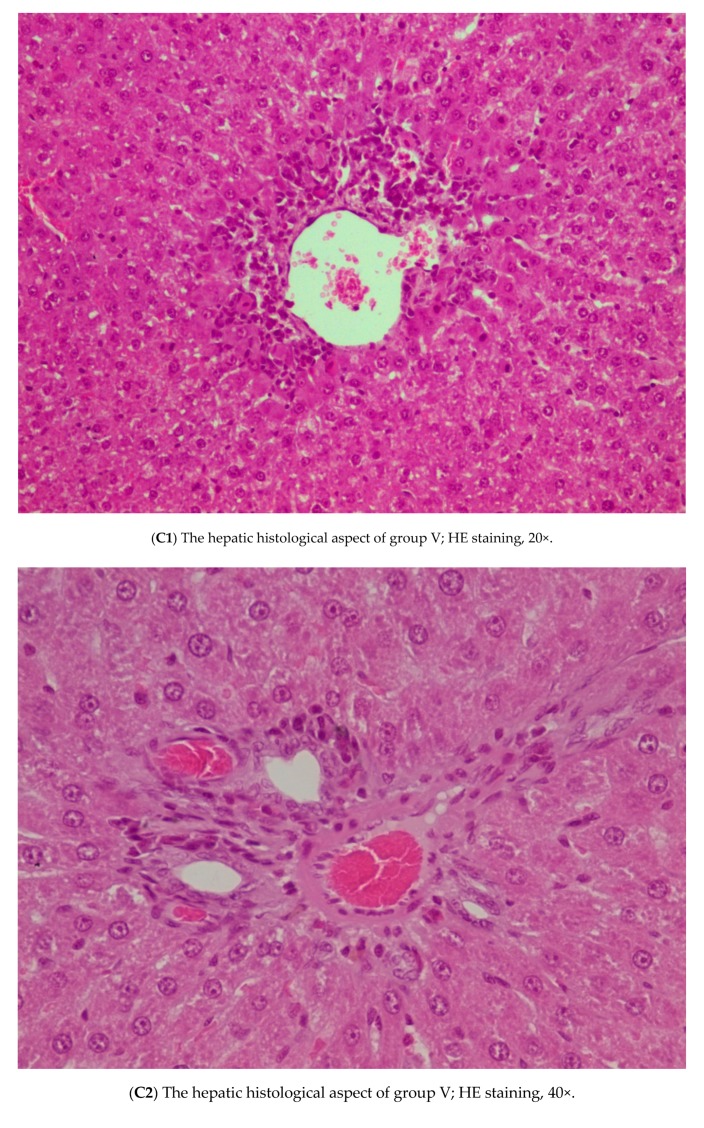

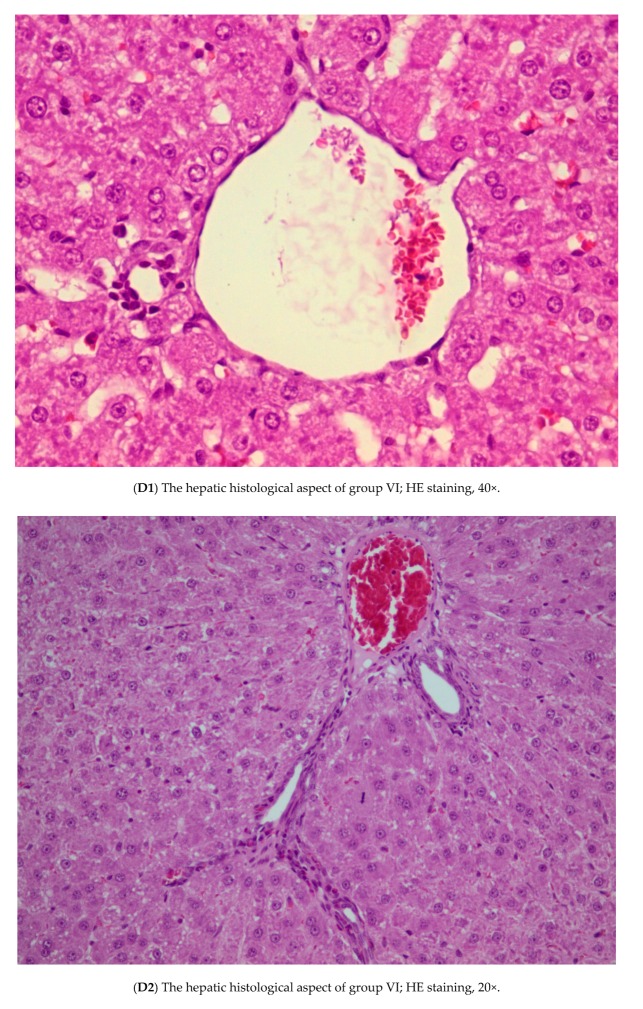

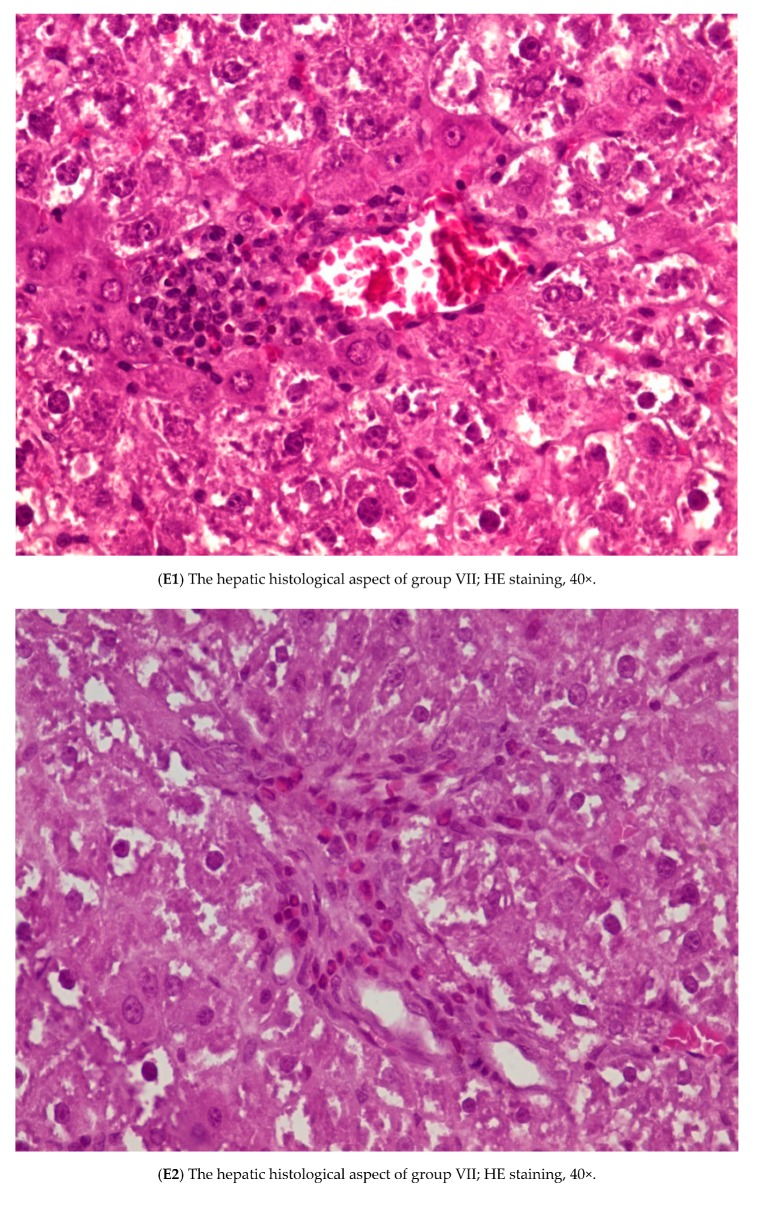
Rats were treated with 1% CMC, APAP, MSB or silymarin either alone or in various combinations (as indicated). Livers were collected after 13 days, formalin-fixed, and embedded in paraffin. Tissue sections were then stained with hematoxylin and eosin and examined for signs of inflammation and liver damage. Control/CMC (group I), (**A**) APAP (group IV), (**B**) APAP + silymarin (group V), (**C**) APAP + MSB (group VI), (**D**) APAP + silymarin + MSB (group VII), (**E**) groups of rats. Abbreviations: APAP, N-acetyl-p-aminophenol or paracetamol (acetaminophen); CMC, carboxymethylcellulose; MSB, MegaSporeBiotic™.

Examination of the histological architecture of the livers from groups I, II and III revealed that the tissue was normal (Figure 4A).

Examination of liver sections in group IV showed several histological changes in the liver structure. Focal hepatocellular necrosis, porto-central necrotic bridges (25% of the sectional area) and diffuse and circumferential pericentral hepatitis (all portal spaces) were observed in these livers (Figure 4B1,B2). 

In group V, 11 of the 33 central veins examined presented adjacent hepatitis with around 2/3 of the circumference involved. Minimal perivenular hepatocyte dystrophy was observed in approximately 10% of the sectional area. All portal spaces had low eosinophil and lymphocyte inflammatory infiltrates and were without interface hepatitis (Figure 4C1,C2).

Liver sections from group VI showed that 2 of the 46 central veins viewed had mild adjacent hepatitis. Also, of the 13 portal spaces identified on the whole section, 6 had a low-level of eosinophilic and lymphocytic inflammation. A single portal space with mild interface hepatitis was identified. A single focus of lobular hepatitis was identified for the entire sectional area. Hepatocyte dystrophy was absent (Figure 4D1,D2).

In group VII, 50% of the central veins presented mild perivenular inflammation with eosinophils and lymphocytes, and 20% had moderate inflammation associated with perivenular hepatitis. Also, hepatocyte dystrophy was observed in about 20% of the sectional area and over 70% of portal spaces had few eosinophils and lymphocytes and no interface hepatitis was observed (Figure 4E1,E2).

Pretreatment with silymarin (group V), MSB (group VI) or both (group VII) significantly prevented changes in hepatic parenchyma compared to group IV, which was treated with APAP alone.

### 3.4. Effect of MegaSporeBiotic™ and Silymarin on Tight Junction

**Figure 5:**
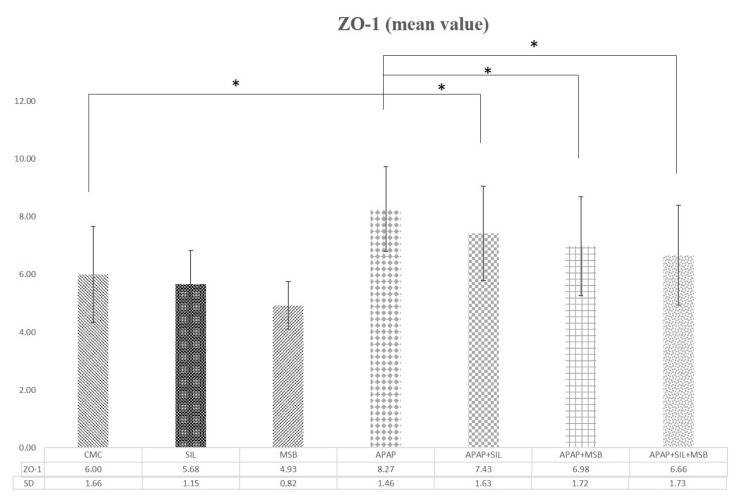
Changes in tight junction protein (ZO-1) levels. Rats were treated with 1% CMC, SIL, MSB, APAP, APAP + SIL, APAP + MSB or APAP + SIL + MSB. Periorbital blood was collected on Day 13 and the serum was tested for ZO-1 levels. * Significant difference (*p* < 0.05) between groups; data were analyzed using one-way ANOVA. Abbreviations: APAP, N-acetyl-p-aminophenol or paracetamol (acetaminophen); CMC, carboxymethylcellulose; MSB, MegaSporeBiotic™; SIL, silymarin; ZO-1, zonula occludens.

The tight integrity of the junction was evaluated by quantifying the expression of ZO-1, a major TJ protein. ZO-1 increased significantly after APAP administration, and silymarin and MSB significantly reduced its level (Figure 5).

## 4. Discussion

In this study, we examined, for the first time, the effect of probiotic spores (MSB) on acute hepatic injury induced by APAP in rats. APAP-induced hepatotoxicity is a classic and well known experimental model that is used to evaluate the hepatoprotective activity of nutraceuticals. Biochemical data obtained in the present study demonstrated that MSB pretreatment ameliorated APAP-induced acute liver injury. Also, histopathological hepatic injury was improved by preadministration of MSB or silymarin versus the disease model group (APAP alone).

As expected, there were no differences between the groups I–III (control, silymarin and MSB) in terms of liver enzyme levels (AST, ALT) (*p* > 0.05 vs. group IV). However, significant differences were observed between the APAP group (group IV) and groups pretreated with silymarin, MSB or both (groups V, VI and VII, respectively); AST and ALT levels were significantly reduced in groups V, VI and VII compared to group IV.

Regarding histopathological aspects, no changes were observed in groups I–III, but hepatic necrosis occurred in livers from the APAP group (group IV, positive control). Significant improvements (vs. group IV) were observed in rats pretreated with silymarin (group V), MSB (group VI) or both (group VII). However, the pretreated MSB group (group VI) had the best hepatic protection (vs. groups V and VII). Structural changes in the liver sections were minimal.

APAP-induced hepatotoxicity is associated with oxidative stress in the liver. APAP is metabolized to a reactive (toxic) metabolite, NAPQI, which is efficiently detoxified by GSH, a nonenzymatic antioxidant (an antioxidant molecule with a low-molecular-weight) that acts as a free-radical scavenger [20]. Administration of APAP in high doses leads to a high amount of NAPQI followed by GSH depletion [21]. GSH deficiency increases the production of reactive oxygen species (ROS), and consequently, oxidative stress in the liver [22]. Cell lesions (inflammation, apoptosis) and then cell death occurs due to oxidative stress [4]. Our study reported that APAP overdose causes TAC to slowly decrease compared to the control group, but pretreatment with silymarin and MSB increases TAC levels. There are no differences between APAP groups and silymarin compared to APAP and MSB. 

Although there is not much information about probiotics and their antioxidant and hepatoprotective effects, it is known that *Bacillus* sp produce exopolysaccharide (ESP) and has significant antioxidant and immunoregulatory activities. EPS production by *B. coagulans* has significant in vitro antioxidant activity [23,24]. Moreover, Duc et al. has shown that *Bacillus* spore-forming bacteria have the ability to produce carotenoids [25], which have strong antioxidant properties. This effect is very important in humans because they are not able to synthesize carotenoids [26]. Rana et al. demonstrated that carotenoid supplementation increases GSH levels (in liver and blood) in experimentally induced hepatotoxicity in rats. Increased levels of GSH induced by carotenoids indicate the protection of hepatocytes in rats with drug-induced hepatotoxicity [27]. Most likely, MSB is able to restore the balance between ROS and antioxidants through the contained *Bacillus* sp and as such, reduces APAP-induced liver damage. *B. subtilis* HU36 is a well-studied, unique, patented, Gram-positive, spore-forming bacteria strain that produces a distinct yellow–orange pigmentation. The pigmentation is due to the synthesis of carotenoids; which are gastric stable, bio-accessible, and significantly more bioavailable than carotenoids from other sources, such as lycopene, lutein, astaxanthin, zeaxanthin and beta-carotene; as well as essential vitamins B and K2 [28].

Leaky gut syndrome is characterized by the dysfunction or disruption of the intestinal barrier. When this happens, toxins such as endotoxins (lipopolysaccharide; LPS) are translocated to the lamina propria and may subsequently lead to many diseases [29]. Duysburgh et al. showed that *Bacillus* spores coadministered with prebiotics in a symbiotic formula, significantly influenced the microbial activity in the intestine, increasing the production of colonic butyrate. This is an important short-chain fatty acid that helps retain the structure of the intestinal barrier and blocks aberrant expression of ZO-1, thus decreasing endotoxemia [30]. 

Tight junction (TJ) proteins are pivotal structures in maintaining the function of the mucosal barrier. It has been shown that ZO-1 expression increased after liver injury (ischemia/reperfusion) and butyrate reversed this aberrant expression. Thus, butyrate may have a protective effect on TJ proteins after reperfusion injury with intestinal congestion [31].

In the current study, we found elevated plasma concentrations of ZO-1 in the groups of rats treated with APAP overdose compared with groups without APAP or with those receiving APAP in combination with silymarin, MSB or both. The changes were positively correlated to levels of other proinflammatory cytokines (TNF-α, IL-1*β*). 

A similar correlation between systemic ZO-1 levels and an inflammatory marker (C-reactive protein; CRP) was observed in patients with cirrhosis [32]. Moreover, in intensive care unit patients, the systemic level of ZO-1 was correlated with sepsis severity or multiple organ dysfunction scores [33].

Recently, APAP has been shown to induce disruption of cell-cell TJs in the livers of mice and human hepatic cells, even at low doses [34]. Thus, APAP in high doses may increase endotoxemia, which could, in turn, increase systemic inflammation. It was demonstrated that supplementation with a spore-based probiotic (*Bacillus* sp) reduced the characteristic symptoms of leaky gut syndrome [35] which suggests that our study product (MSB) may strengthen the intestinal barrier and therefore decrease the dissemination of microbial-derived compounds from the intestine (including toxins) due to inflammation-triggered leaky gut, ultimately decreasing endotoxemia. Moreover, by restoring the intestinal barrier, MSB may prevent inflammation.

It is known that once LPS enters the portal vein, it interacts with TLR4 on Kupffer cells. This interaction leads to an elevation of LPS-TLR4–related proinflammatory cytokines. TNF-α is considered the first mediator that is increased, followed by IL-10 and IL-6 [36,37]. Over 20 years ago, Blazka et al. demonstrated a significant increase in serum TNF-α levels after administration of APAP; they also observed that the administration of Kupffer cell inhibitors reduced APAP toxicity in rats [38]. Concerning liver inflammation, MSB decreased the level of proinflammatory cytokines (TNF-α, IL-6) similar to silymarin in APAP-treated animals; the combination of MSB and silymarin reduced TNF-α and IL-6 to normal levels. These results suggest that MSB may also have had an anti-inflammatory effect on rats that were intoxicated with APAP; also, group IV (APAP) presented with severe histopathological hepatic inflammation (necrosis). The other groups pretreated with silymarin, MSB or both (groups V, VI and VII, respectively), exhibited inflammatory eosinophilic infiltrates and lymphocytes either without hepatitis or with mild interface hepatitis. These changes were accompanied by changes in ALT and AST levels. It is likely that MSB has the capacity to change the level of proinflammatory cytokines produced in response to APAP overdose. It was demonstrated that *B. coagulans* decreased the TNF-α similarly to indomethacin in an experimental rat model of rheumatoid arthritis [39], and *B. clausii* inhibited the secretion of proinflammatory cytokines (TNF-α, IL-6, IL-17) and increased levels of anti-inflammatory cytokines (IL-10) in a postmenopausal osteoporotic mouse model [40].

## 5. Conclusions

In conclusion, our study revealed that the probiotic supplement, MSB, which contains *Bacillus* sp spores, had a protective effect on acute hepatic injury induced by APAP overdose in rats. Pretreatment with MSB resulted in a significant reduction in serum AST, ALT, proinflammatory cytokines (TNF-α, IL-1*β*), ZO-1 and TAC, as well as hepatocyte necrosis, which was similar to the well-known hepatoprotective agent, silymarin. These results indicate the hepatoprotective potential of this spore-based probiotic in drug-induced acute hepatotoxicity. It is very important in drug-induced liver toxicity. However, further studies are needed to confirm this in humans.
